# Protection from experimental autoimmune encephalomyelitis by polyclonal IgG requires adjuvant-induced inflammation

**DOI:** 10.1186/s12974-016-0506-x

**Published:** 2016-02-18

**Authors:** Isaak Quast, Christian W. Keller, Patrick Weber, Christoph Schneider, Stephan von Gunten, Jan D. Lünemann

**Affiliations:** Institute of Experimental Immunology, Laboratory of Neuroinflammation, University of Zürich, Zürich, Switzerland; Institute of Pharmacology, University of Bern, Bern, Switzerland

**Keywords:** Intravenous immunoglobulin, Experimental autoimmune encephalomyelitis, Multiple sclerosis, T cell, Dendritic cell, Innate immunity, Adjuvant, Mycobacteria

## Abstract

**Background:**

Intravenous immunoglobulin (IVIG) proved to be an efficient anti-inflammatory treatment for a growing number of neuroinflammatory diseases and protects against the development of experimental autoimmune encephalomyelitis (EAE), a widely used animal model for multiple sclerosis (MS).

**Methods:**

The clinical efficacy of IVIG and IVIG-derived F(ab’)_2_ fragments, generated using the streptococcal cysteine proteinase Ide-S, was evaluated in EAE induced by active immunization and by adoptive transfer of myelin-specific T cells. Frequency, phenotype, and functional characteristics of T cell subsets and myeloid cells were determined by flow cytometry. Antibody binding to microbial antigen and cytokine production by innate immune cells was assessed by ELISA.

**Results:**

We report that the protective effect of IVIG is lost in the adoptive transfer model of EAE and requires prophylactic administration during disease induction. IVIG-derived Fc fragments are not required for protection against EAE, since administration of F(ab’)_2_ fragments fully recapitulated the clinical efficacy of IVIG. F(ab’)_2_-treated mice showed a substantial decrease in splenic effector T cell expansion and cytokine production (GM-CSF, IFN-γ, IL-17A) 9 days after immunization. Inhibition of effector T cell responses was not associated with an increase in total numbers of Tregs but with decreased activation of innate myeloid cells such as neutrophils, monocytes, and dendritic cells. Therapeutically effective IVIG-derived F(ab’)_2_ fragments inhibited adjuvant-induced innate immune cell activation as determined by IL-12/23 p40 production and recognized mycobacterial antigens contained in Freund’s complete adjuvant which is required for induction of active EAE.

**Conclusions:**

Our data indicate that F(ab’)_2_-mediated neutralization of adjuvant contributes to the therapeutic efficacy of anti-inflammatory IgG. These findings might partly explain the discrepancy of IVIG efficacy in EAE and MS.

## Background

High-dose therapy with intravenous immunoglobulin (IVIG), obtained from the plasma of several thousand individuals, is an efficient anti-inflammatory and immunomodulatory treatment for a growing number of autoimmune neuroinflammatory diseases. Based on randomized controlled clinical trials (RCT), FDA-approved indications include chronic inflammatory demyelinating polyneuropathy and multifocal motor neuropathy [1]. IVIG is also effective in some patients with worsening myasthenia gravis and is beneficial as a second-line therapy for dermatomyositis and stiff-person syndrome [1]. IVIG therapy was shown to suppress clinical disease development in experimental autoimmune encephalomyelitis (EAE) [2–8], an animal model for multiple sclerosis (MS). However, subsequent RCTs demonstrated that IVIG therapy has no beneficial effects in reducing relapse rates and clinical disease progression in patients with relapsing-remitting and secondary-progressive multiple sclerosis (MS) [9–12], which led to the conclusion that IVIG is ineffective and not recommended for patients with MS [1, 13, 14].

IVIG preparations contain antibodies directed against a broad range of pathogens, as well as numerous foreign and self antigens. IgG molecules consist of two functional domains, the antigen-binding fragment F(ab’)_2_, which determines the specificity of the antibody molecule, and the constant, or fragment crystallizable (Fc) region, which is critical for the initiation of effector responses such as activation of the complement pathway or crosslinking of Fc-receptors on innate immune effector cells. In autoantibody-mediated animal models of autoimmune diseases such as idiopathic thrombocytopenic purpura (ITP), rheumatoid arthritis, and nephrotoxic nephritis, the IgG Fc domain appears to be essential in mediating the protective effects of IVIG [15–19]. The clinical efficacy of IVIG in EAE, a T cell-driven autoimmune disease model, has been attributed to IVIG-induced inhibition of effector T cells and reciprocal expansion of CD4^+^FoxP3^+^ regulatory T cells (Tregs), but divergent results were obtained on whether the protective effects of IVIG in EAE are Fc-dependent [4, 7, 8]. Here, we show that the protective effect of IVIG is dose-dependent, requires prophylactic administration during priming, and is lost upon adoptive transfer of encephalitogenic T cells to induce EAE. IVIG-derived F(ab’)_2_ fragments inhibit immunostimulatory adjuvant activity necessary for disease induction and are sufficient to mediate protection from EAE development.

## Methods

### Purification of Ide-S

The protein sequence of Ide-S (NCBI reference sequence: NP_269065.1; AA28-339) was reverse translated into an *E. coli* codon-optimized DNA sequence using CLC Main Workbench (Qiagen). The resultant sequence was synthesized by GeneArt^TM^ gene synthesis (Thermo Fischer Scientific) and subcloned in a modified pET28a (GE Healthcare) vector containing an N-terminal deka-HIS tag. MC1060/pWTZ594 *E. coli* was used for cloning and plasmid amplification. The final plasmid was transferred into BL21 *E. coli* (NEB). For protein expression, bacteria were grown to an OD_600 nm_ of 0.3–0.4 and expression was induced by addition of 0.1 mM IPTG (AppliChem) for 3 h at 37 °C. The bacteria pellet was suspended in PBS containing 20 μg/ml DNAse (Sigma) and 1.6 mM PMSF. Bacteria were lysed by sonication and Ide-S was purified by immobilized metal ion affinity chromatography (HisTrap HP columns, GE Healthcare) using Äkta prime plus (GE Healthcare). Successful purification was monitored by SDS-PAGE and Coomassie® Brilliant Blue R250 staining. Finally, the protein was dialyzed to PBS, sterile filtered through a 0.2 μM filter, supplemented with 20 % glycerol and adjusted to a concentration of 1 mg/ml before snap-freezing in liquid nitrogen and storage at −80 °C until further use.

### Generation of F(ab’)_2_ fragments from IVIG

The streptococcal cysteine proteinase Ide-S was used to generate F(ab’)_2_ fragments from IVIG [20]. Privigen (CSL Behring) was used as IVIG preparation throughout the study. Two milligrams of Ide-S were incubated with 3 ml (300 mg) IVIG at room temperature (RT) for at least 8 h (or overnight). F(ab’)_2_ was separated from uncut IgG and Fc using a HiLoad 26/60 Superdex 75 prep grade column (GE Healthcare) and Äkta Purifier (GE Healthcare) using PBS as running buffer. The F(ab’)_2_ containing fraction was concentrated by ammonium sulfate precipitation by adding twice the volume of saturated ammonium sulfate solution and incubation for 1 h at RT. After centrifugation for 30 min at 3000×*g*, the supernatant was discarded and the precipitate suspended in PBS and extensively dialyzed to PBS. To remove remaining full-length IgG, a protein A sepharose (GE Healthcare) column was self-packed by filling 2 ml protein A sepharose slurry in a Supelco polypropylene SPE tube containing PE frits with 20 μM porosity (Sigma-Aldrich) and F(ab’)_2_ was applied by gravity flow. The column flow-through, containing the F(ab’)_2_, was concentrated using Amicon Ultra 15-ml centrifugal filters (Merck Millipore) with a molecular weight cutoff of 10 kDa according to the manufacturer’s instructions until a final concentration of 35 to 45 mg/ml was obtained. Successful purification of F(ab’)_2_ was confirmed by SDS-PAGE and Coomassie® Brilliant Blue R250 staining. Finally, the solution was sterile-filtered through a 0.2-μM filter and stored at 4 °C until further use.

### Mice

C57Bl/6 wt mice (B6) were purchased from Janvier. C57Bl/6 TCR^MOG^ transgenic mice (2D2) mice were kindly provided by Vijay K. Kuchroo (Harvard Institutes of Medicine) [21]. 2D2 mice were genotyped as previously described [21]. All mice were bred and housed in the animal facility of the University of Zürich in individually ventilated cages under specific pathogen free conditions according to Swiss animal laws. All animal protocols were approved by the cantonal veterinary office of Zürich, Switzerland (license numbers 862012 and 702015).

### Induction and assessment of active EAE

EAE was induced in B6 mice as previously described [22, 23] with minor adaptations. In brief, MOG_35–55_ peptide (MEVGWYRSPFSRVVHLYRNGK; GenScript) was dissolved in PBS to obtain a final concentration of 1 mg/ml. On the day of immunization, 100 μl peptide solution was emulsified in 100 μl complete Freund’s adjuvant (CFA; Difco Laboratories) supplemented with 3.3 mg/ml dried, inactivated *Mycobacterium tuberculosis* (*M. Tuberculosis* Des. H37 Ra, Difco Laboratories, Detroit, USA) by vigorously mixing the solution for 15 min via transfer in between two syringes connected to each other by a Luer-Lock connector. Six- to eight-week-old female B6 mice or TCR^MOG^ transgenic mice were used for immunization. Mice were anesthetized by isoflurane inhalation and immunized by s.c. injection of 100-μl emulsion on both sides of the lateral abdomen using a 24 G × 1” needle. In addition, mice received 200 ng pertussis toxin (pertussis toxin in Glycerol, List Biological Laboratories) i.p. on the day of immunization and 2 days thereafter. Animal weight and general health and disease progression were monitored daily. The following scoring system was applied: 0—no detectable signs of EAE; 0.5—distal limp tail paralysis; 1.0—complete limp tail paralysis; 1.5—limp tail paralysis and hindlimb weakness; 2.0—unilateral partial hindlimb paralysis; 2.5—bilateral partial hindlimb paralysis; 3.0—complete bilateral hindlimb paralysis; 3.5—complete bilateral hindlimb paralysis and partial forelimb paralysis; 4.0—moribund; 5.0—dead. Mice were euthanized by CO_2_ inhalation if a disease score of 3 was maintained for more than 7 days, a disease score of 3.5 was maintained for more than 3 days, or a disease score of 4 was reached.

### Induction and assessment of adoptive transfer EAE

Adoptive transfer EAE was induced as previously described [24, 25]. Donor mice (2D2) were immunized with MOG_35–55_ peptide emulsified in CFA as described above. On day 7 post immunization, leukocytes from the spleen and draining lymph nodes were purified (see below). Cells were restimulated in vitro by cultivation for 2 days at a density of 1 × 10^7^ cells/ml at standard cell culture conditions (SCCC; 37 °C and 5 % CO_2_ in a humidified incubator) in 12-cm cell culture dishes (Greiner) in RPMI 1640 medium (Life Technologies; 10 ml per dish) supplemented with 50 U/ml penicillin/streptomycin (P/S) and 10 % fetal calf serum (FCS) (referred to as R10 medium), 10 ng/ml recombinant IL-23 (eBioscience), and 20 μg/ml MOG_35–55_ peptide. Recipient mice were sublethally irradiated with 550 rad 1 day before i.p. injection of 1 × 10^7^ restimulated cells. Disease progression was monitored as described above.

### Leukocyte isolation from spleen and lymph nodes

Mice were euthanized by CO_2_ inhalation. Spleen and inguinal lymph nodes were removed and mechanically disrupted using scissors. Disrupted tissues were incubated at 37 °C in 3 ml PBS containing 0.4 mg/ml Collagenase D (Roche) and 0.1 mg/ml DNA se (Sigma) for 30 min. The reaction was stopped by adding EDTA to a final concentration of 10 mM. Thereafter, a syringe plunger was used to further disrupt the tissue by filtering through a 70-μm mesh. Cells were washed once with PBS. Red blood cells (RBC) were lysed using RBC lysis buffer (Biolegend) according to the manufacturer’s instructions. CASY® counter (Innovatis) was used to determine cell numbers.

### Flow cytometry analysis of murine splenocytes

For the analysis of cell surface antigens, 2 × 10^7^ cells were suspended in 50 μl PBS supplemented with fluorochrome coupled monoclonal antibodies. After incubation on ice for 30 min, cells were washed twice with 200 μl PBS. For intracellular cytokine staining, 2 × 10^7^ cells were incubated per well of a 96-well V bottom plate in 100 μl R10 supplemented with 50 ng/ml PMA (Sigma), 500 ng/ml Ionomycin (Sigma), and 10 μg/ml Brefeldin A (Sigma) for 4 h at SCCC. For intracellular detection of cytokines and transcription factors, FoxP3 transcription factor staining buffer set (eBioscience) was used according to the manufacturer’s instructions. All samples were acquired using LSR Fortessa (BD). Fluorochrome labeled antibodies were purchased from Biolegend (anti-CD8α-FITC or anti-CD8α-BV785 (clone 53-6.7), anti-CD11b-APC-Cy7 (clone M1/70), anti-CD11c-PE-Cy7 (clone N418), anti-CD25-BV605 (clone PC61), anti-CD44-Pacific Blue (clone IM7), anti-CD80-PE (clone 16-10A1), anti-IL-17A-PE-Cy7 (clone TC11-18H10), anti-IFN-γ-FITC (clone XMG1.2), anti-Ly6C-PerCP -Cy5.5 (clone HK1.4), anti-Ly6G-APC (clone 1A8), and anti-MHC-II-Pacific Blue (I-A/I-E, clone M5/114.15.2)), eBioscience (anti-CD19-Alexa-Fluor700 (clone eBio1D3 (1D3), anti-FoxP3-PerCP-Cy5.5 (clone FJK-16s), and anti-GM-CSF-PE (clone MP1-22E9)), and BD Pharmingen (anti-CD4-APC (clone RM4-5) and anti-CD62L-PE-CF594 (clone MEL-14)). Fixable L/D staining kits (aqua/amcyan and near-IR) were purchased from Invitrogen.

### Analysis of neutrophil cell death

Mouse bone marrow-derived neutrophils were isolated from B6 mice as previously described [26]. Cell death was assessed using annexin V/propidium-iodide (PI) and DNA fragmentation assays by flow cytometry (FACSVerse; Becton Dickinson Biosciences), as previously described [27, 28]. PI was purchased from Sigma-Aldrich (Buchs, Switzerland). Recombinant hexahistidine-tagged GFP-annexin V, Fas-Ligand (Fas-L) and Fas-L cross-linker were kind gifts from Prof. T. Kaufmann, Institute of Pharmacology, University of Bern, Bern, Switzerland.

### In vitro stimulation of splenocytes with *M. tuberculosis*

Mouse splenocytes were isolated as described above. 5 × 10^5^ splenocytes were plated in 96-well U-bottom plates (Greiner) in R10 medium and incubated in the presence or absence of F(ab’)_2_ fragments with 0.001, 0.01, and 0.1 μg/ml dried, inactivated *M. tuberculosis* (Des. H37 Ra, Difco Laboratories) suspended in R10 medium for 12 h under SCCC.

### IL-12/23 p40 ELISA

IL-12/23 p40 was detected in culture supernatants by ELISA using the monoclonal antibodies C15.6 and C17.8 according to the manufacturer’s recommendations (Mouse IL-12/23 (p40) ELISA; Mabtech).

### *M. tuberculosis* binding ELISA

Dried, inactivated *M. tuberculosis* (Des. H37 Ra, Difco Laboratories, Detroit, USA) was suspended and diluted to a final concentration of 50 mg/ml in 50 mM carbonate buffer (pH 9.6). Per well, 50 μl diluted *M. tuberculosis* were coated overnight at 4 °C to flat-bottom 96-well plates (MaxiSorp®, Nunc). After washing extensively with PBS containing 0.1 % Tween20 (wash buffer), wells were blocked with PBS containing 1 % bovine serum albumin and 0.05 % Tween20 (blocking buffer) for 2 h at RT. Thereafter, all liquid was removed, and IVIG, F(ab’)_2_, or Rituximab (Mabtera, Roche) were diluted to 1, 0.1, 0.01, and 0.001 μg/ml with blocking buffer and added to the wells. After incubation for 1 h at RT, plates were washed with wash buffer and incubated for 1 h with biotinylated mouse-anti-human IgG (BD, clone G18-145) diluted in blocking buffer to a final concentration of 0.5 μg/ml. Wells were washed again and incubated with horseradish peroxidase (HRP) coupled streptavidin (Mabtech). 1-StepTM Ultra TMB-ELISA Substrate Solution (ThermoFischer Scientific) was used to detect HRP activity. The reaction was stopped by addition of 2 M NaCl, and colorimetric signals were evaluated by measuring the optical density at 450 nm.

### Statistics

Statistics were performed using GraphPad Prism 5 (GraphPad Software Inc.). *p* values below 0.05 were considered significant.

## Results

### Clinical efficacy of IVIG in EAE requires high-dose treatment and active immunization

**Fig. 1:**
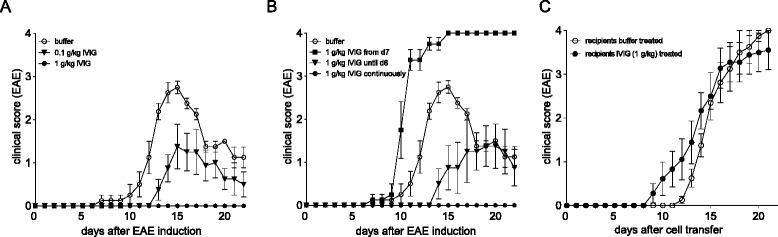
Efficacy of IVIG in active vs. adoptive transfer EAE. B6 mice were immunized with MOG_35–55_ peptide emulsified in CFA and treated i.p. with IVIG or PBS starting 1 day before immunization and every day thereafter (or as indicated). Titration (**a**) and timing (**b**) of IVIG treatment. Means and SEM are shown. *n* = 4 animals per group. **c** EAE was induced by adoptive transfer of in vitro restimulated leukocytes derived from MOG_35–55_ and CFA immunized 2D2 mice. Mice received daily i.p. injections of IVIG or buffer starting 1 day before immunization. Means and SEM are shown. *n* = 10 animals per group

We first evaluated the IVIG dose required for its protective effect. IVIG was given daily starting 1 day before EAE immunization. High-dose IVIG (1 g/kg) completely protected animals from disease development whereas 0.1 g/kg only led to partial protection (Fig. 1a). Continuous administration of IVIG prevented EAE development, termination of treatment at day 6 led to a delayed disease onset and reduced severity and treatment from day 7 on exacerbated EAE symptoms (Fig. 1b) indicating that IVIG is required at high dose and continuously for its protective effect and active during T cell priming in response to active vaccination. In order to investigate the therapeutic potential of IVIG independent of adjuvant administration and T cell priming, we next determined its clinical efficacy in the adoptive transfer model of EAE, in which disease is induced by peripheral introduction of pre-activated myelin-specific effector T cells. Spleen and lymph node (LN)-derived leukocytes from MOG_35–55_ peptide immunized mice carrying the transgenic 2D2 T cell receptor recognizing MOG_35–55_ peptide [21] were restimulated in vitro with IL-23 and MOG_35–55_ peptide and adoptively transferred into naïve mice. High-dose IVIG treatment (1 g/kg) in recipient mice was started 1 day before transfer and continued throughout the experiment. In contrast to its therapeutic efficacy in active EAE, IVIG treatment was ineffective in protecting recipient mice from disease development following adoptive transfer of myelin-specific T cells (Fig. 1c). These data show that IVIG protects from EAE development if administered during disease induction following active immunization but does not significantly inhibit the encephalitogenic potential of primed effector T cells.

### Fc fragments are not required for the protective effect of IVIG in EAE

**Fig. 2:**
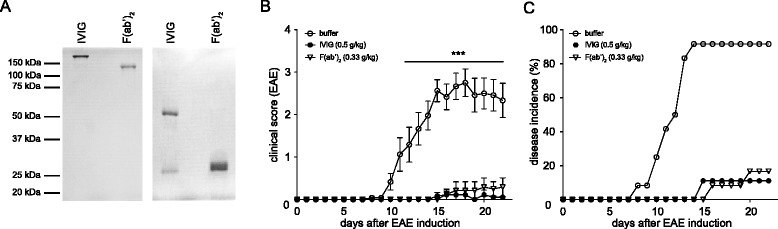
Fc is not required for the protective effect of IVIG. **a** Coomassie® Brilliant Blue stained non-reducing (*left*) or reducing (*right*) SDS-PAGE showing IVIG and purified F(ab’)_2_. B6 mice were immunized with MOG_35–55_ peptide emulsified in CFA and injected i.p. with IVIG, F(ab’)_2_, or buffer starting 1 day before immunization. Disease severity (**b**) and incidence (**c**) are shown. *n* = 12 animals per group. Individual curves in (**b**) were compared to buffer treated animals using two-way ANOVA and Bonferroni posttest. ***p ≤ 0.001

To determine whether the protective effect of IVIG in EAE requires the presence of Fc fragments as reported for several antibody-mediated autoimmune disease models [15–19], the Fc portion of IVIG-derived IgG was removed by cleavage with Ide-S [20] and F(ab’)_2_ was purified. Successful purification was confirmed by SDS-PAGE and Coomassie® Brilliant Blue staining (Fig. 2a). EAE-immunized mice were treated with equimolar amounts of IVIG, F(ab’)_2_, or buffer starting 1 day before immunization. F(ab’)_2_ fragments completely recapitulated the protective effect of IVIG for both disease severity (Fig. 2b) and incidence (Fig. 2c) indicating that Fc fragments are not required for the protective effect of IVIG during EAE development.

### IVIG-derived F(ab’)_2_ fragments inhibit expansion and cytokine production of activated effector T cells

**Fig. 3:**
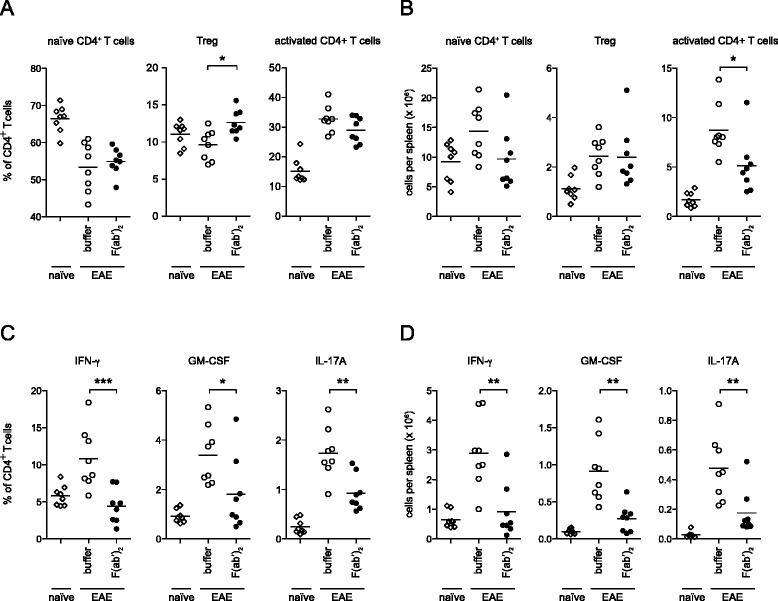
IVIG-derived F(ab’)_2_ fragments inhibit effector T cell differentiation and cytokine production. B6 mice were either left untreated (naïve) or immunized with MOG_35–55_ peptide emulsified in CFA and injected i.p. with F(ab’)_2_ (0.33 g/kg) or buffer starting 1 day before immunization and every day thereafter. Analysis of splenic CD4^+^ T cells 9 days after immunization is shown. Percentage (**a**) and absolute numbers (**b**) of the indicated CD4^+^ T cell subsets. Frequency (**c**) and absolute numbers (**d**) of CD4^+^ T cells producing IFN-γ, GM-CSF, or IL-17A following restimulation with PMA/ionomycin. Statistics were performed by Mann Whitney test. *p ≤ 0.05, **p ≤ 0.01, ***p ≤ 0.001

In order to determine T cell regulatory mechanisms responsible for the protective effect of IVIG during disease induction, we analyzed frequencies, absolute numbers, and cytokine production of CD44^−^CD62L^+^FoxP3^−^ naïve, FoxP3^+^ regulatory, and CD44^+^FoxP3^−^-activated effector CD4^+^ T cells in F(ab’)_2_-treated and non-treated mice 9 days after immunization. F(ab’)_2_ treatment was initiated 1 day prior to immunization and maintained until day 9. As reported previously [4], F(ab’)_2_ treatment was associated with increased frequencies of splenic Tregs (Fig. 3a). In absolute numbers, however, F(ab’)_2_ did not expand Tregs but decreased the numbers of activated CD4^+^ T cells (Fig. 3b). Furthermore, F(ab’)_2_-treated mice showed substantially reduced frequencies and absolute numbers of CD4^+^ T cells producing INF-γ, GM-CSF, or IL-17A (Fig. 3c, d). These data indicate that the protective effect of IVIG-derived F(ab’)_2_ fragments is associated with a marked reduction in activated effector T cells producing cytokines crucial for the development of EAE [25, 29].

### Reduced phenotypic innate immune cell activation upon F(ab’)_2_ treatment in vivo

**Fig. 4:**
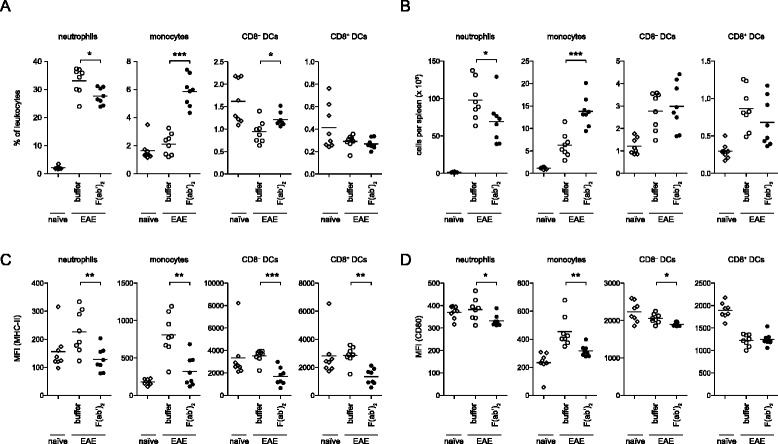
F(ab’)_2_ treatment impairs innate immune cell activation. B6 mice were either left untreated (naïve) or immunized with MOG_35–55_ peptide emulsified in CFA and injected i.p. with F(ab’)_2_ (0.33 g/kg) or buffer starting 1 day before immunization and every day thereafter. Analysis of splenic neutrophils, monocytes, and dendritic cell subsets 9 days after immunization is shown. Frequencies (**a**) and absolute numbers (**b**) of the indicated leukocyte subset and cell surface expression levels of MHC class II (**c**) and CD80 (**d**). *MFI* median fluorescence intensity. Statistics were performed by Mann Whitney test. *p ≤ 0.05, **p ≤ 0.01, ***p ≤ 0.001

Induction of active EAE following immunization requires administration of adjuvant such as complete Freund’s adjuvant (CFA)-contained inactivated mycobacteria, e.g., *M. tuberculosis*, leading to innate immune cell activation and effector T cell expansion [30–32]. To evaluate whether IVIG-derived F(ab’)_2_ fragments inhibit innate immune cell activation in response to immunization, we analyzed frequencies of leukocyte subsets (Ly6G^+^CD11c^int^ neutrophils, Ly6C^high^CD11b^+^ monocytes and CD11c^high^MHC class II^+^ CD8^−^ or CD8^+^ DCs) and profiled their expression of cell surface markers associated with activation and antigen presentation (MHC class II, CD80). F(ab’)_2_ treatment affected the composition of innate leukocytes leading to reduced numbers of neutrophils and increased numbers of monocytes 9 days after immunization (Fig. 4a). In all of the innate immune cell subsets analyzed, F(ab’)_2_ treatment led to a marked reduction of MHC class II and CD80 expression (Fig. 4b), indicating that the protective effect of IVIG-derived F(ab’)_2_ fragments is associated with both reduced innate immune activation and limited expansion of effector T cells.

### F(ab’)_2_ treatment does not induce apoptosis in murine neutrophils

**Fig. 5:**
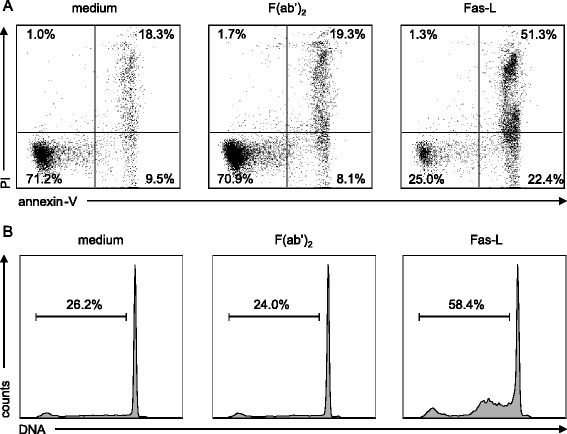
IVIG-derived F(ab’)_2_ fragments do not induce cell death of murine neutrophils. Flow cytometric analysis of cell death by annexin V-FITC/PI staining (**a**) or DNA fragmentation (**b**) of cells cultured in presence of 13.3 mg/ml F(ab’)_2_ or 110 ng/ml crosslinked Fas-L (positive control). Results of 15-hour cultures are shown. Data are representative of three independent experiments. Quantitative analysis is indicated as the percentage representative of each quadrant (**a**) or of gated subdiploid DNA (**b**)

Neutrophils have been shown to play an important role during EAE disease initiation as they can amplify autoimmune CNS infiltrates by maturing local antigen-presenting cells and neutrophil depletion ameliorates EAE development [33]. IVIG was shown to contain Fas and Siglec-9 specific antibodies, which can induce apoptosis in human neutrophils [28, 34, 35]. In murine neutrophils, IVIG (supplied by the Japan Blood Products Organization, Osaka, Japan; 0.3 and 1 g/kg, administered intraperitoneally) was reported to limit inhibition of neutrophil apoptosis induced by lipopolysaccharide (LPS) stimulation, potentially by blocking LPS-mediated effects, although no direct pro-apoptotic activity of IVIG could be demonstrated [36]. We therefore examined whether F(ab’)_2_ fragments induce apoptotic cell death in murine neutrophils as a potential mechanism contributing to the protective effect of IVIG during EAE development. Apoptotic cell death was determined by annexin V, PI staining, and DNA fragmentation in *ex vivo* cultured neutrophils in the presence of 13.3 mg/ml F(ab’)_2_, a concentration which has been reported to readily induce apoptosis of human neutrophils [28] and crosslinked Fas-L as positive control (Fig. 5). Murine neutrophils were resistant to apoptotic cell death induced by human F(ab’)_2_ fragments arguing against F(ab’)_2_-mediated cytotoxicity of neutrophils as a potential mechanism of IVIG efficacy in EAE.

### IVIG-derived F(ab’)_2_ fragments recognize mycobacterial antigens in CFA and inhibit mycobacterial-induced IL-12/23 p40 production

**Fig. 6:**
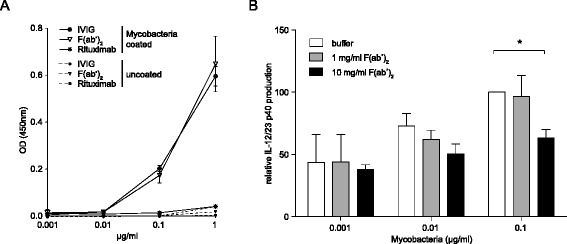
IVIG-derived F(ab’)_2_ fragments bind to CFA-contained mycobacterial antigens and inhibit *Mycobacteria*-induced IL-12/23 p40 production in vitro. **a** Binding of IVIG, IVIG-derived F(ab’)_2_, or Rituximab to Mycobacteria-coated ELISA plates. **b** Murine splenocytes were incubated overnight with mycobacteria in the presence or absence of F(ab’)_2_. IL-12/23 p40 secretion into culture supernatants was measured by ELISA. IL-12/23 p40 production in the presence of 0.1 μg/ml mycobacteria in the absence of F(ab’)_2_ was set to 100 %, and relative IL-12/23 p40 production was calculated for each experiment. Mean + SEM of three independent experiments is shown. Statistics were performed by paired *T* test. *p ≤ 0.05

Decreased innate immune cell activation in response to CFA/MOG_35–55_ peptide-immunization during F(ab’)_2_ treatment suggests that IVIG might contain antibodies specific for mycobacterial antigens which could reduce the immunostimulatory potential of CFA and, thus, impair the immunogenicity of EAE immunization. Indeed, bacterial antigens are recognized by IVIG [37, 38], and IVIG was reported to be therapeutically active in *M. tuberculosis* infection in mice [39]. To determine whether IVIG-derived F(ab’)_2_ fragments bind to CFA-contained mycobacterial antigens, the mycobacterial fraction of CFA was coated to ELISA plates and incubated with IVIG, IVIG-derived F(ab’)_2_, and a control monoclonal antibody (Rituximab). Both IVIG and IVIG-derived F(ab’)_2_ fragments readily bound mycobacterial antigens (Fig. 6a). We next determined whether IVIG-derived F(ab’)_2_ treatment would inhibit the immunostimulatory efficacy of the mycobacterial fraction of CFA to elicit IL-12/23 p40 production, a cytokine crucial for EAE development [31, 40]. Challenge of murine splenocytes with dried, inactivated *M. tuberculosis* led to a dose-dependent increase in IL-12/23 p40 secretion, which was inhibited in the presence of IVIG-derived F(ab’)_2_ fragments (Fig. 6b). These data indicate that IVIG contains CFA-binding antibodies and inhibits the immunostimulatory activity of CFA-containing mycobacterial antigens required for induction of active EAE.

## Discussion

We demonstrate that high-dose IVIG treatment protects from EAE development if administered during disease induction. These findings are in line with a previous study demonstrating that IVIG is only effective in EAE if administered prophylactically but does not attenuate the disease course or the degree of CNS inflammation if administered after onset of symptoms [41]. In the aforementioned study, brain and cervical spinal cord inflammation tended to be higher in mice that received IVIG at day 8–9, i.e., after onset of clinical symptoms. In our hands, IVIG treatment of actively induced EAE exacerbated disease if administered continuously after day 7. Myelin-specific IgG does not induce but exacerbates EAE if administered into already diseased mice [42] indicating that pathogenic antibodies can accelerate but not induce EAE development. The observed exacerbation of EAE pathology following IVIG administration after day 7 suggests that IVIG indeed contains IgG species that could promote disease development such as antibodies targeting myelin structures or neutralizing anti-inflammatory mediators [43–45], but the exact mechanisms responsible for this finding remain to be identified.

Although IVIG was reported to enter the CNS and is localized to inflammatory lesions during EAE [6], we show that its therapeutic efficacy is lost in the adoptive transfer EAE model indicating that IVIG interferes with early stages of disease development but does not inhibit already primed and pre-activated encephalitogenic T cells or inflammatory lesion formation within the CNS.

Here, Privigen (CSL Behring) was used throughout the study. Different IVIG preparations are frequently treated as interchangeable products clinically, but differences in product preparations exist that may impact tolerability and activity in selected applications [46]. Further studies are required to determine whether our findings are applicable to other IVIG preparations as well. Our study, however, extends the findings by Achiron and colleagues who reported that IVIG treatment with Gamimune N (Bayer), while therapeutically beneficial in EAE induced with guinea pig myelin basic protein in complete Freund’s adjuvant, had no effect in relation to onset, duration, and severity in the adoptive transfer EAE model in Lewis rats [47].

IVIG preparations were shown to suppress the proliferation of human myelin- and tetanus toxoid-specific T cells (Octagam, Octapharma and Venimmun, Sanofi-Aventis) [48], to induce apoptosis in human leukemic cells of lymphocyte and monocyte lineage (Gammagard, Baxalta, and Sandoglobulin, CSL Behring) [49] and to confer anergy to T cells specific for myelin basic protein (Sandoglobulin, CSL Behring) [50]. The lack of efficacy in preventing EAE development in mice following adoptive transfer of myelin-specific T cells argues against a function of IVIG in inhibiting the viability or expansion of pathogenic effector T cells in vivo. Moreover, it does not support a major role for IVIG in promoting oligodendrocyte apoptosis during EAE as discussed by Weishaupt and colleagues (Venimmun, Sanofi-Aventis and Sandoglobulin, CSL Behring) [51] nor in promoting myelin repair as suggested by Humle Jorgensen et al. [5] since oligodendrocyte injury and demyelination contribute to the development of both active and adoptive transfer EAE. This being said, our data do not exclude the possibility that other IVIG preparations than the ones tested in our study (Privigen) and by Achiron et al. [47] (Gamimune N) might be effective in limiting adoptive transfer EAE development.

The anti-inflammatory efficacy of IVIG has been attributed to both F(ab’)_2_- and Fc-dependent mechanisms [52]. Fc fragments appear to be required for IVIG-mediated protection in several antibody-mediated autoimmune disease models such as the K/BxN serum transfer arthritis model and the antiplatelet monoclonal antibody 6A6-mediated model for immune thrombocytopenia [15, 18, 53, 54]. In contrast to EAE, the aforementioned autoimmune disease models are largely mediated by IgG-dependent activation of innate immune effector cells via crosslinking of cellular FcγRs [17, 55–68]. Antibodies raised by the immunization with MOG peptide during EAE induction do not facilitate disease susceptibility, as mice lacking B cells develop severe EAE [69–71]. Mice deficient in the FcR-associated signaling unit, the common γ-chain, are EAE resistant; however, the protective effect of FcγR deficiency was shown to be dependent on complement activation but independent of antibodies and immune complexes binding to FcγR expressing cells [72, 73]. The finding that treatment with F(ab’)_2_ fragments strongly reduced effector T cell numbers and pro-inflammatory cytokine production and fully phenocopied the protection achieved with equimolar amounts of IVIG indicates that F(ab’)_2_ fragments are the key in mediating the clinical efficacy of IVIG in EAE.

A prime example of an F(ab’)_2_-dependent mechanism is toxic epidermal necrolysis (Lyell syndrome), in which the Fas–FasL (also called CD95–CD95L) receptor–ligand pathway is a crucial mediator for the skin blistering associated with this condition. By blocking this interaction, CD95-specific antibodies in IVIG preparations can interfere with this autoimmune pathology [74]. The human immune repertoire recognizes a broad range of antigens which is reflected by the finding that IVIG preparations contain antibodies with specificity not only for foreign, microbial antigens but also for host factors such as attachment sites for viral and bacterial pathogens [38]. For example, IVIG binds to glycans important for host-pathogen interactions [38] and is reported to contain antibodies binding to pertussis toxin [75, 76], which is used along with CFA to facilitate active EAE induction.

Our study shows that antibodies binding to mycobacterial antigens are present in IVIG preparations, inhibit the immunogenicity of CFA necessary for EAE induction, and ameliorate disease development. These findings might partly explain the discrepancy between the clinical efficacy of IVIG in the EAE model which requires active immunization with microbial adjuvant [2–8] and MS patients where IVIG has limited efficacy in reducing relapse rate and disease progression.

## Conclusions

IVIG proved to be an efficient anti-inflammatory treatment for a growing number of neuroinflammatory diseases and protects against the development of actively induced EAE. Although EAE is a widely used animal model for MS, there is an apparent discrepancy in the clinical efficacy of IVIG in MS and EAE. Using one IVIG preparation (Privigen), we found that the protective effect of polyclonal IgG is lost in the adoptive transfer model of EAE and requires prophylactic administration during disease induction. IVIG-derived Fc fragments are not required for protection against EAE, since administration of F(ab’)_2_ fragments fully recapitulate the clinical efficacy of IVIG. F(ab’)_2_-mediated protection of actively induced EAE is associated with decreased innate immune cell activation and effector T cell responses upon vaccination. As a potential mechanism, we demonstrate that IVIG binds to mycobacterial antigens in Freund’s complete adjuvant which is required to facilitate active EAE induction. These findings might partly explain the discrepancy of IVIG efficacy in MS and the EAE model which requires active immunization with microbial adjuvant.
